# A Posteriori Comparison of Natural and Surgical Destabilization Models of Canine Osteoarthritis

**DOI:** 10.1155/2013/180453

**Published:** 2013-10-31

**Authors:** Maxim Moreau, Jean-Pierre Pelletier, Bertrand Lussier, Marc-André d'Anjou, Laurent Blond, Johanne-Martel Pelletier, Jérôme R. E. del Castillo, Eric Troncy

**Affiliations:** ^1^Osteoarthritis Research Unit, Université de Montréal Hospital Centre, Notre-Dame Hospital, 1560 Sherbrooke St. East, Montreal, QC, Canada H2L 4M1; ^2^GREPAQ, Department of Veterinary Biomedical Sciences, Faculty of Veterinary Medicine (FVM), Université de Montréal, P.O. Box 5000, Saint-Hyacinthe, QC, Canada J2S 7C6; ^3^Department of Clinical Sciences, FVM, Université de Montréal, P.O. Box 5000, Saint-Hyacinthe, QC, Canada J2S 7C6; ^4^Hôpital Vétérinaire Rive-Sud, 7415 Taschereau, Brossard, QC, Canada J4Y 1A2; ^5^Clinique Vétérinaire Languedocia, 395 Ruc Maurice Béjart, 34080 Montpellier, France

## Abstract

For many years *Canis familiaris*, the domestic dog, has drawn particular interest as a model of osteoarthritis (OA). Here, we optimized the dog model of experimental OA induced by cranial cruciate ligament sectioning. The usefulness of noninvasive complementary outcome measures, such as gait analysis for the limb function and magnetic resonance imaging for structural changes, was demonstrated in this model. Relationships were established between the functional impairment and the severity of structural changes including the measurement of cartilage thinning. In the dog model of naturally occurring OA, excellent test-retest reliability was denoted for the measurement of the limb function. A criterion to identify clinically meaningful responders to therapy was determined for privately owned dogs undergoing clinical trials. In addition, the recording of accelerometer-based duration of locomotor activity showed strong and complementary agreement with the biomechanical limb function. The translation potential of these models to the human OA condition is underlined. A preclinical testing protocol which combines the dog model of experimental OA induced by cranial cruciate ligament transection and the Dog model of naturally occurring OA offers the opportunity to further investigate the structural and functional benefits of disease-modifying strategies. Ultimately, a better prediction of outcomes for human clinical trials would be brought.

## 1. Introduction

Biomedical research is the broad area of science that investigates the biological processes and the causes of diseases mainly through experimentation and testing. Enticing this vision, the use of animal models is required to advance medical knowledge and overall health benefits. In the field of rheumatic diseases such as osteoarthritis (OA), animal models contribute to the understanding of the basic biology of OA and help to develop potent therapeutic approaches for the benefits of human medicine [1]. Unfortunately, a consensus regarding the ideal animal model for studying OA has not been established [2–4]. Actually, there is a need to optimize current models of OA and to propose avenues to enhance preclinical drug development.

The canine stifle is similar to a human's knee, sharing anatomical components and histological aspects [5]. To give a deep insight in the OA mechanisms, the dogs have been subjected to several approaches over the years to induce the structural changes of OA, including cartilage scarification (or groove model) [6], transarticular impact [7], tibial osteotomy [8], and meniscal lesions [9]. Another well-described dog model of OA is the cranial (or anterior) cruciate ligament (CCL) transection. Surgical CCL transection (CCLT) alters the amount and distribution of biomechanical forces. Over days to months, the joint features structural changes that mimic OA, including synovitis, osteophyte growth, cartilage depletion, and bone marrow lesions (BMLs) development [10].

The conventional scientific outputs (i.e., joint structural changes) of the experimental dog model of OA induced by CCLT have been recently coupled to peak vertical force (PVF) measurement using kinetic gait analysis to document concomitant potential benefits on the pain-related functional impairment [11–13]. The first aim of this study was to optimize the experimental CCLT-induced dog OA model by further exploring the translational relationship between the level of structural changes and the limb disability. 

Developmental arthropathies and joint trauma predispose dog to structural changes of OA, which like in human beings lead to crippling pain and disability [14–16]. The potential of pharmaceutical as well as complementary and alternative medicines has been tested in different randomized controlled trials (RCTs) in naturally occurring OA dogs using PVF as an outcome measure of pain-related functional impairment [17–20]. Naturally occurring models of OA have been proposed even to accelerate the development of human therapeutics [10]. As a second aim, this study would characterize different outcome measures in a manner to optimize the use of naturally occurring OA dogs in research and to improve the quality of RCT in this translational natural model.

## 2. Materials and Methods

### 2.1. Dog Model of Experimental OA

#### 2.1.1. Specific Research Objectives

**Figure 1:**
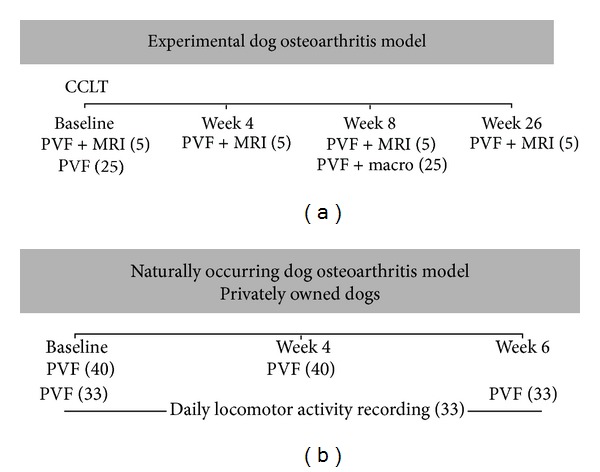
Schematic view of the data reported in (a) the dog model of experimental osteoarthritis and (b) the dog model of naturally occurring osteoarthritis. PVF (peak vertical force), MRI (magnetic resonance imaging), macro (macroscopic structural measurement of cartilage thinning), CCLT (cranial cruciate ligament transection). Numbers of dogs are specified in parenthesis.

The evolution of the PVF measurement and its relationship with the progression of structural changes evaluated on magnetic resonance imaging (MRI) scans (i.e., cartilage volume loss, focal changes of the articular cartilage, BMLs, osteophytes, joint effusion size, and meniscal lesions) was documented over a period of 26 weeks in CCL-deficient dogs. In addition, the relationship between PVF recording and the macroscopic measurements of cartilage thinning performed at eight weeks following CCLT was documented cross-sectionally. Such relationship served to determine the level of *in vivo* structural changes to be predicted based on a given PVF measurement. To this end, data were selected from previous studies involving PVF measurement and structural changes on MRI (internal data, 2005) [21–23] and macroscopic measurement of cartilage thinning (internal data 2005, 2007) [11, 12] (Figure 1(a)). 

 All experiments were approved by the Institutional Animal Care and Use Committee in accordance with the guidelines of the Canadian Council on Animal Care. All dogs were acclimated, housed, and then subjected to surgical CCLT of the right knee under preemptive (transdermal fentanyl 50 or 75 *μ*g/h; Janssen Ortho, Markham, ON, Canada) and multimodal (intra-articular block combined with opioid administration) analgesia as previously described [11]. Food was given once daily and removed overnight. Body weight was monitored weekly and was kept constant throughout the study duration. Throughout the study, all dogs were actively exercised in exterior runs (1.35 m × 9.15 m) for a 2-hour period, 5 days a week, under the supervision of an animal care technician.

#### 2.1.2. Peak Vertical Force Measurement

Recognized as a reference method of functional outcome in dog [24–26], the PVF measurement was done at the trot (1.9–2.2 meter/second) using a floor mat-based plantar force measurement system (Walkway with four Matscan sensors 3150; Tekscan Inc., Boston, MA, USA), as previously described [11]. Data were acquired at four successive time points (Figure 1(a)). For the CCL-deficient hind limb, the first stride PVF was acquired. Data from the first five valid trials were averaged and expressed as a percentage of body weight (% BW) and used to describe the change over time in PVF measurement.

#### 2.1.3. Magnetic Resonance Imaging

Structural changes were evaluated at four successive time points (Figure 1(a)) using MRI scans (Echospeed LX; General Electric Healthcare, Waukesha, WI, USA) and settings as previously described [21]. Previous publications detailed the quantification of cartilage volume (mm^3^) [21] and the scoring system used for focal changes of the articular cartilage (0–4, maximum score of 44) [21], BMLs (0–3, maximum score of 27) [23], osteophytes (0–3, maximum score of 45) [22], joint effusion size (0–3) [22], and meniscal lesions (0–3, maximum score of 6) [27]. Cartilage volume and structural changes were evaluated using the following sequences: (1) three-dimensional spoiled gradient recalled sequence (SPGR) with fat suppression, (2) T1-weighted three-dimensional fast gradient recalled echo (T1w-GRE), and (3) T2-weighted fast spin echo sequence with fat saturation (T2w-FS). Bone marrow lesions were scored independently in T1w-GRE and T2w-FS sequences as ill-defined areas of hypointensity or hyperintensity, respectively. Evaluation of cartilage volume and structural changes was for the entire (global) joint, except otherwise stated. 

#### 2.1.4. Macroscopic Measurement of Cartilage Thinning

Cartilage thinning was quantified at eight weeks following CCLT (Figure 1(a)) using a dissecting microscope (Stereozoom; Bausch & Lomb, Rochester, NY, USA) as previously described [28]. Macroscopic measurement of cartilage thinning (mm^2^) was for the medial and lateral femoral condyles and medial and lateral tibial plateaus.

### 2.2. Dog Model of Naturally Occurring OA

#### 2.2.1. Specific Research Objectives

The repeatability, standard error of measurement (SEM), and minimal detectable change (MDC) of PVF measured in privately owned dogs affected by naturally occurring OA were defined over a four-week period. Moreover, the PVF measurement was tested for its relationship with accelerometer-based duration of daily locomotor activity. The goal of testing such relationship was to determine the level of PVF measurement exceeding the MDC to be predicted based on a change in daily locomotor activity, which would represent a practical outcome of animal welfare determinant at home [29]. To this end, data were selected from previous studies involving PVF measurement (40 placebo-treated dogs followed up over four weeks) [17, 19, 30] or PVF measurement coupled to daily locomotor activity recording over six weeks (33 dogs, from which 14 were placebo-treated) (internal data, 2007) [17] (Figure 1(b)). All studies were approved by the Institutional Animal Care and Use Committee in accordance with the guidelines of the Canadian Council on Animal Care. All owners gave informed consent for their participation in each RCT.

#### 2.2.2. Naturally Occurring OA Dogs

Seventy-three privately owned adult dogs weighing more than 20 kg having radiographic evidence of OA exclusively at the hip and/or stifle joints were considered, as previously described [17, 19, 30]. All dogs had OA-related hind limb disability according to orthopedic examinations and PVF measurements. Specific washout periods were respected for eventual OA treatment (including pharmaceuticals, natural health products, and therapeutic diets).

#### 2.2.3. Peak Vertical Force Measurement

The PVF measurement was done at the trot (1.9–2.2 meter/second) using a force platform (Model OR6-6, Advanced Mechanical Technology Inc, Watertown, Massachusetts, USA), as previously described [17, 19, 29, 30]. Measurements were done at different time points (Figure 1(b)). Averaged data from the first five valid trials were expressed as % BW. In each dog, the hind limb with the lowest PVF measurement was used for statistical analyses purpose.

#### 2.2.4. Daily Locomotor Activity Recording

Accelerometer-based daily locomotor activity recording was done using Actical system (Bio-Lynx Scientific Equipment Inc., Montreal, QC, Canada) as previously described [17]. Collar-mounted accelerometers were worn by 33 dogs for six weeks, 24 hour/day (Figure 1(b)). The duration of motion was continuously monitored as counts every two minutes, giving 720 counts per day. Daily duration of active period was referred to the time spent (expressed in minutes) when the count exceeded 30 in terms of intensity. This cut-off value was based on internal data (2004) in comparison to video-analysis and was previously used to discern movement in active (intensity > 30) from inactive (intensity < 30) period [17]. Data used were the area under the curve (AUC) which represents the integral of the daily duration of active period over six weeks and the mean of the first three days, and of the last seven days, which defined Baseline and week six data, respectively [31].

### 2.3. Statistical Analyses

To describe the evolution of limb function in CCL-deficient dogs, a Friedman test was used using Dunn's tests for *post hoc* analyses. To describe the relationship between the limb function with MRI structural changes, data were analyzed with Spearman correlation test and presented as Spearman coefficient (*r*_*s*_). This coefficient shows by its magnitude the strength of the linear association. An *r*_*s*_ close to one (or minus one) indicates a strong positive (negative) linear correlation. To describe the relationship between the limb function (explanatory variable) with macroscopic measurement of cartilage thinning (response variable), data were analyzed with mixed linear model using studies as random effect. Random-effect models attempt to generalize findings beyond the included studies by assuming that the selected studies are random samples from a larger population. Such models incorporate a component of between-study variation into the uncertainty of the estimates [32]. The general equation of the linear regression was *y* = *mx* + *b*, where *m* refers to the slope and *b* refers to the *y*-intercept (i.e., the value of *y* when *x* = zero). To describe the natural fluctuation in limb function of placebo-treated (negative control) privately-owned dogs, absolute reliability (test-retest) was calculated using intraclass coefficient of correlation (ICC) and related 95% confidence intervals (95% CI). Two-way random single measures model (ICC 2.1) was used. An ICC close to one indicates “excellent” reliability [33]. The SEM quantifies the precision of individual PVF measurement and defines the boundaries around which a subject's value is expected to lie according to a given confident interval [34]. The SEM at 95% CI was calculated as follows:

(1) $$\begin{array}{c} \text{SEM}=\text{SD}*\sqrt{1}-\text{ICC}, \end{array}$$

where SD referred to the within-subject standard deviation [35]. The MDC in PVF measurement that can be recorded confidently (95% CI) between test sessions is referred to as the MDC_95_ and was calculated as follows:

(2) $$\begin{array}{c} \text{MD}{\text{C}}_{95}=\text{SEM}*1.96*\sqrt{2}. \end{array}$$

The MDC_95_ can be interpreted as the magnitude of change, below which there is more than a 95% chance that change has occurred as a result of measurement error [36]. Outside this change, value does reflect a real alteration in the functional impairment toward improvement or worsening in privately-owned dog with naturally occurring OA. To describe the relationship between the limb function (response variable) with daily locomotor activity recording (explanatory variable), data were analyzed with mixed linear model using study arms (placebo or tested agent) as fixed factor and studies as random factor. All analyses were performed with SPSS, version 20.0 (SPSS Inc., Chicago, IL, USA). Values are presented as mean (standard deviation). Significant level was set at *P* < 0.05.

## 3. Results

### 3.1. Dog Model of Experimental OA

#### 3.1.1. Measurement of the PVF

**Figure 2:**
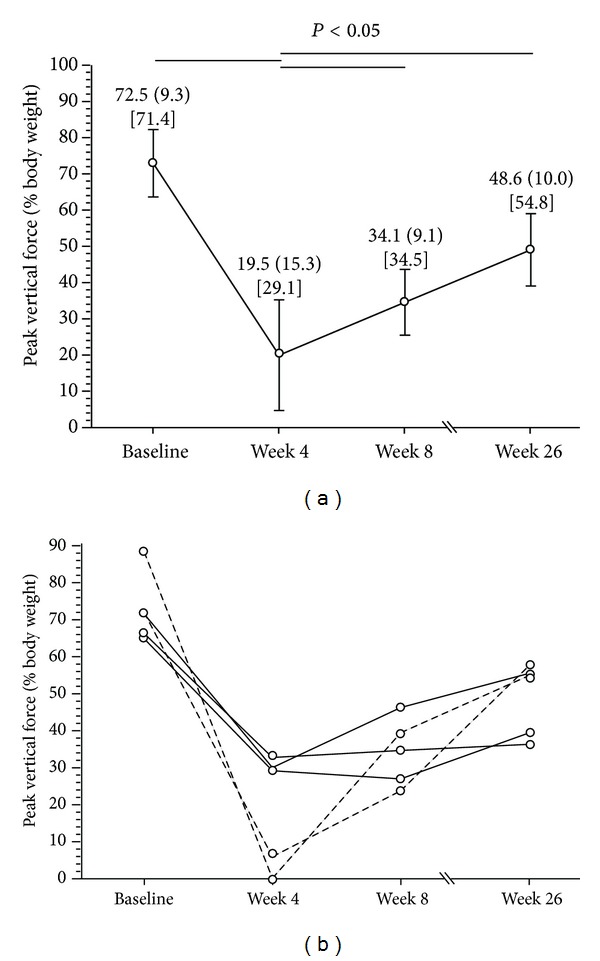
(a) Averaged peak vertical force values measured before (Baseline) and four, eight, and 26 weeks after cranial cruciate ligament transection in dogs. At each time point, group values are presented as mean (standard deviation) (median). Dunn's test identified which medians were significantly different. (b) Individual peak vertical force values measured before (Baseline) and four, eight, and 26 weeks after cranial cruciate ligament transection in dogs. Dotted lines identify dogs having the highest limb disability at week four and the highest levels of focal changes of the articular cartilage.

Peak vertical force measurement changed over time (*P* < 0.003) following CCLT (Figure 2(a)). Based on medians, there was a significant decrease at week four and at week eight when compared to Baseline. Then, PVF increased at week 26, reaching values significantly different than week four only. The individual changes over time involved different degrees of functional impairment characterized by a decrease in PVF measurement from Baseline reaching a nadir (at week four) followed by a phase of remission (from week four to week 26, Figure 2(a)).

#### 3.1.2. Relationship between PVF and Structural Changes on MRI

During the phase of functional impairment nadir (from Baseline to week four), the decrease in PVF measurement did not correlate in a significant manner with the development of structural changes as evaluated using MRI (Table 1). Of note, the dogs having the more severe limb disability at week four (Figure 2(b)) were those with the highest level of focal changes of the articular cartilage.

**Figure 3:**
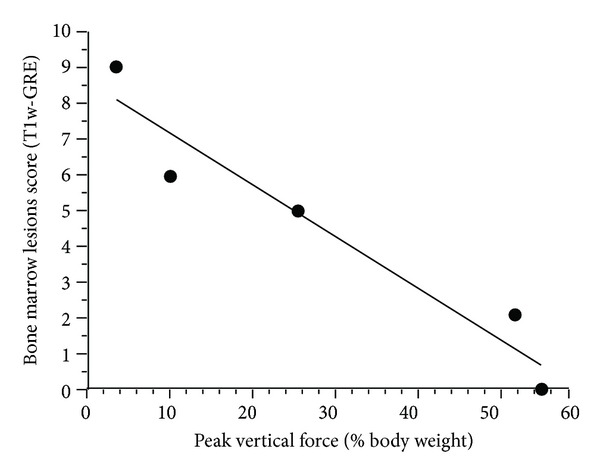
Significant correlation (*r*_*s*_ = −0.99,  *P* < 0.001) for the differences of hypointense bone marrow lesions on T1-weighted three-dimensional fast gradient recalled echo images (T1w-GRE) scores during the remission phase (week 26 minus week four), with the concurrent difference in peak vertical force measurement. Linear regression trend is illustrated.

The increase in PVF measured during the phase of remission (from week four to week 26) correlated inversely with the score for osteophytes, joint effusion, hypointense BMLs (Figure 3) and focal changes of the articular cartilage (Table 1). These negative correlations mean an abrogated remission in the presence of severe chondral and subchondral lesions and MRI-scored joint effusion.

The measurement of PVF did not correlate with cartilage volume loss, hyperintense BMLs, or meniscal tears. Only a trend was seen for a positive correlation with medial cartilage volume loss (the more PVF remission was, the more cartilage volume loss was) and medial tears score of the meniscus (Table 1).

#### 3.1.3. Relationship with Macroscopic Measurement of Cartilage Thinning

Twenty-five dogs undergoing PVF measurement before and after CCLT were used. At Baseline, PVF measurement was 70.4 (10.9)% BW and was 26.6 (12.4)% BW and 33.9 (15.8)% BW at week four and eight, respectively. The PVF measured at week eight did not demonstrate significant relationship with cartilage thinning observed on the lateral condyle and plateau and medial plateau (Table 2). However, a significant relationship was observed with the severity of the thinning at the medial condyle, which means higher PVF value in the presence of more severe cartilage thinning. According to the regression parameters (see Table 2), for a group of CCL-deficient dogs weighing 25.0 (2.3) kg, a PVF measured at week eight of 33.9 (15.8)% BW is expected to correspond to an extent of cartilage thinning on the medial condyle of 27.3 mm^2^ (95% CI: 10.4–44.2).

### 3.2. Dog Model of Naturally Occurring OA

#### 3.2.1. Characteristics of PVF Measurement

**Figure 4:**
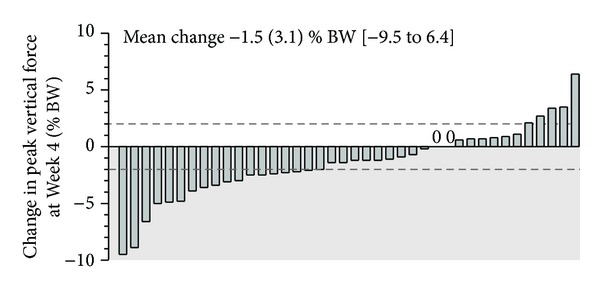
Individual changes in peak vertical force measured at week four in 40 privately owned dogs receiving a placebo in randomized controlled trials. Changes were the difference between week four versus Baseline. Grey zone represents a decrease in peak vertical force measurement compared to Baseline. Dashed lines represent the MDC_95_. Peak vertical force data are expressed in % BW (body weight) and presented as mean (standard deviation) (minimal value to maximal value).

Forty privately owned dogs affected by OA who received a placebo (negative control) served to determine the test-retest reliability of PVF measurement over a period of four weeks (Table 3). Standard error of measurement was determined and served for the calculation of MDC_95_. The MDC_95_ was consistent with an increase or a decline in the magnitude of 2.0% BW across this group of OA dogs. When expressed relatively to Baseline value, the MDC_95_ represented 3.6%.  Figure 4 presents individual changes in PVF measured from Baseline to week four. According to the MDC_95_, 22 dogs had clinically meaningful changes, which were positive in five dogs and negative in 17 others. 

#### 3.2.2. Relationship with Daily Locomotor Activity Recording

Thirty-three privately-owned dogs affected by OA that had PVF measurement and daily locomotor activity recorded over a six-week period were used. The PVF measurement in OA dogs demonstrated a significant relationship with the integral (AUC) of the daily duration of active period recorded during the 26-week period (*P* = 0.001), which means a higher PVF in the presence of higher locomotor activity. The change in the PVF measurement demonstrated a significant relationship with the change in daily duration of active period (*P* = 0.003, *m* = 0.03 (95% CI: 0.01–0.05), *b* = 2.8 (95% CI: 0.4–5.1)) regardless of the study arms (i.e., placebo or tested agents). According to the regression parameters, for an increase in daily duration of active period by 54 minutes in OA dogs, the change in PVF measurement was predicted to be 4.4% BW (95% CI: 2.1–6.8). This by far exceeds the previously defined MDC_95_ (i.e., 2.0% BW), meaning a significant positive effect in PVF measurement (limb function) related to the increase in locomotor activity.

## 4. Discussion

### 4.1. Dog Model of Experimental OA

The CCLT dog model of OA involves structural changes that mimic those encountered in human OA [2, 10, 37]. This model was further optimized keeping in mind the three Rs' principles of replacement, reduction, and refinement [38]. The present study demonstrated the usefulness of complementary outcome measures. Hence, PVF measurement, which echoes pain-related functional impairment, can be successfully combined to the common structural outcomes in the CCLT dog model of OA. To maximize the information gained from CCL-deficient dogs, researchers can document in a noninvasive manner the pain-related disability, which comprises a phase of functional impairment with a nadir preceding a remission process. At the preclinical stage of drug development, such information has a clinically meaningful potential for disease-modifying compounds proposed to confer pain and functional improvement in addition to structural benefits. As PVF measurement data were detailed, power and sample size can be determined *a priori*, again supporting the principle of reduction by providing statistical estimates. The principle of refinement is also addressed by documenting individual variability (per dog data) that occurs over an extended duration [39]. 

Anterior cruciate ligament (human counterpart of CCL) transection leads to rotational and translational changes that induce mechanical stresses on articular surfaces unaccustomed for such loading solicitation [40–44]. This phenomenon generates loss of tissue integrity, involving abnormal architecture and components known as structural changes. Using MRI, which is a non-invasive imaging technique, the present study supports the use of the CCLT dog model of OA for its potential to corroborate the relationship between structural changes and clinical signs observed in human. Hence, a recent systematic review indicated that OA knee pain is associated with BMLs and effusion/synovitis [45]. In line with findings observed in humans [46, 47], severe limb impairment was denoted in dogs with the highest level of focal changes of the articular cartilage during the phase of limb disability (from Baseline to week four). When BMLs evolved minimally, these manifestations were concomitant to lesser limb disability (Figure 3). Such benefits are in accordance with the report of Zhang et al. [48] who observed a fluctuation of pain when BMLs were modulated. We also observed that CCL-deficient dogs had better limb functional remission when osteophyte growth and joint effusion size were minimal. These findings were suggestive of higher pain in the presence of osteophyte and effusion/synovitis as reported in human [45, 49, 50]. In face of the present results, we propose to integrate the pain-related functional impairment to the presence of severe chondral and subchondral lesions. We suggest that a more unstable joint (i.e., devoid of adaptive neuromuscular strategies to palliate for the excessive displacement) could be responsible for the more severe chondral and subchondral changes observed. As an attempt to restore limb function, marked expressions of secondary strategies, such as osteophytes growth and joint effusion, are suggested to develop for providing stability and cushioning, in a manner to minimize the deleterious knee joint load in CCL-deficient dogs.

Although we did not reach statistically significant levels, our findings are suggestive of a role of mechanical environment in cartilage volume loss and meniscus insult in CCL-deficient dogs. Hence, we found a trend to have more severe cartilage loss and meniscal tears in the medial part of the joint in dogs having recovered well their limb function (i.e., with the highest PVF measurement). In addition, the extent of medial cartilage thinning was greater in dogs having the highest limb function at eight weeks following CCLT. Those findings were in line with those of Smith jr. et al. [13], which reported a link between the level of knee joint chondropathy and increasing limb function in this model. Furthermore, meniscal lesions have been linked with the progression of OA cartilage loss in humans [51, 52] while a strong relationship exists between high joint loading and meniscal lesions [53]. These findings are of major importance, not only because of their correspondence to findings in human knee OA, but also because the presence of meniscal lesions has an impact on the response to disease-modifying OA drug (DMOAD) treatment in human knee OA [46, 51], a finding that likely also applies to the CCLT dog model of OA.

Despite its burgeoning importance, translation of DMOAD therapies from the laboratory into clinical practice has slowed. Differences between the OA models studied preclinically and the disease evaluated in human clinical trials contribute to this failure [54]. First, a general concern is the use of quadruped animals as knee models for the bipedal human, particularly given their range of motion differences noted in a study comparing large animal (cow, sheep, goat, dog, pig, and rabbit) to human cadaveric knees [55]. The disappearance of many of the observed differences in the cruciate and meniscal anatomy after normalization with the tibial plateau width suggested an overall conservation of relative size among species for the cruciates and menisci [55]. This anatomical and biomechanical analogy, while reviewing the different OA animal models, led Gregory et al. [3] to state that the canine model is probably the closest to a gold-standard animal model for OA currently available. The present study adds the structure-function relationship translated from the dog CCLT model to the human pain OA condition.

Second, most animal models of OA induce disease through chemical insult or surgical or mechanical disruption of joint biomechanics in young individuals rather than the spontaneous development of the disease. This instability-induced joint disease in animals best models the structural changes that develops in humans after an injurious event, known as posttraumatic OA [54]. The poor translational predictability to therapy response is particularly high with the rodent preclinical models. Studies in genetically modified mice suggest that post-traumatic OA has a distinct molecular pathophysiology compared with that of spontaneous OA, which might explain the poor translation from preclinical to clinical OA therapeutic trials [54]. On the contrary, molecular changes observed in a past study with the canine CCLT model suggest that dog cartilage responds to post-traumatic OA and degenerative conditions by regulating the same genes in a similar direction as that observed for chondrocytes in post-traumatic and late human OA [56]. Finally, the recent finding about the DMOAD effects of strontium ranelate [57] late in the CCLT dog model of OA being confirmed in a Phase III clinical trial in knee OA patients [58] is of the utmost importance in the context of this publication. Previously, many DMOADs have demonstrated efficacy in the dog OA model, including the matrix metalloproteinase inhibitor doxycycline [59, 60], the viscosupplementation *via* local hyaluronan [61, 62], the antiresorptive agents such as bisphosphonate [11, 63] and calcitonin [64], the anti-inflammatory properties of diacerhein [65], licofelone [28], and NSAIDs (such as carprofen). All these products but calcitonin (probably related to a deficient formulation) demonstrated similar efficacy in human OA [51, 66–73]. To the best of authors' knowledge, no other preclinical animal OA model presents a better translational predictability record, partly because species differences with respect to the relative contribution of various mediators, receptors, or enzymes to the pathology and xenobiotics metabolism are common.

In accordance with the three Rs' principles, the predictive character of the cartilage thinning based upon PVF measurement opens the idea of limiting the requirement of post-mortem analysis for future research aimed to gain insight in joint cartilage integrity in this model. Based on the regression parameters, the limb disability observed at eight weeks following CCLT predicted an extent of macroscopic lesions surface by 27.3 mm^2^. This level of lesions represents 12% of the total surface of the medial condyle when based on MRI cartilage surface mapping in dogs of similar BW (ArthroVision, personal communication, 2013). As the characterization of full-thickness cartilage thinning in end-stage OA in humans was shown to range between 10 and 23% at this joint compartment [74], the translational potential (macroscopical structural argument) of this model to human OA is further supported.

It should be pointed out that the statistical method used to correlate structural changes on MRI with PVF measurement does not pinpoint the sequence of events and did not take into account the potential role of confounding factors, interrelationship, and dependency. Findings of the pilot study reported herein will help to promote future research of a more mechanistic (structure-function) approach based on a higher sample size. The complementary outcome measures proposed herein to optimize the use of the dog in OA research are not restricted to the CCLT model. Other experimental avenues should be explored for their potential to induce structural changes in close relationship with functional impairment.

### 4.2. Dog Model of Naturally Occurring OA

The recent interest in natural models of OA [10] puts more emphasis on the need to improve the rigor of RCT using functional outcome measures, such as PVF, in naturally occurring OA dogs. This study optimized the use of naturally occurring OA dogs in research by characterizing the PVF measurement with regards to the high value of this outcome to address pain/biomechanics-related joint alterations in the dog. Here test-retest PVF measurement values demonstrated excellent between-session reliability with an ICC of 91 (95% CI: 80–95) in placebo-treated dogs followed up over a four-week period. The SEM provides an absolute index of reliability and refers to the precision of individual measurements. Determining magnitude of an intervention benefit is a critical methodological step in the design of a clinical trial. For the PVF, the MDC_95_ indicated that a change of at least 2.0% BW needs to occur to be confident, at the 95% level, that a change in PVF measurement reflects a real change and not a difference that is within what might be reasonably expected given the measurement error (noise). As PVF measurement characteristics were provided, such as standard deviation, SEM, and the MCD_95_, such data should help researchers to estimate power and sample size, thus contributing to the principle of refinement. 

Randomized controlled trials in naturally occurring OA dogs usually focus on testing mean changes across groups of treated (test article) and control (placebo-treated) animals. This practice often obscures the individual change, which may be very informative in clinical studies [75]. Moreover, reporting the percentages of subjects who met the MDC_95_ requirements provides additional insightful interpretations other than considering only the overall mean change scores [76]. Accordingly, researchers have a tool to distinguish improved (or worsened) dogs by using the proposed MDC_95_ value as a cutoff. Of note, the level of 2.0% BW was in line with the improvement observed following therapeutic modalities in previous clinical RCT in OA dogs [17–20, 30, 77–80]. 

The results of the current study show that different levels of change in limb function reflected by PVF measurement were observed in privately-owned dogs afflicted by OA (Figure 4). Among the 40 dogs evaluated, 22 (55%) had clinically meaningful changes, which were positive (placebo effect) in five (12.5%) or negative (nocebo effect) in 17 (42.5%) dogs. The high proportion of dogs having a worsening of their condition contributes mainly to the overall decrease in PVF recording by −1.5 (3.1)% BW. A phenomenon known as the maturation effect may be suggested as being involved in changes exceeding the measurement error.

In a recent multicenter RCT in naturally occurring OA, an arbitrary cut-off value (i.e., 2.8% BW or ≥5% of Baseline measurement) was used to distinguish clinically meaningful responders from measurement error [81]. Of note, the global rate of responders reported according to this value was 20.7% whereas it was higher in our study (55%) by applying the MDC_95_ (calculated to be 2.0% BW or ≥3.6% of Baseline measurement). The latter finding is important, as applying the higher arbitrary cut-off value rather than the MDC_95_ proposed herein would lead to a high false negative rate of responders (being indeed considered as nonresponders). This type II error overestimates the required sample size and leads to an unnecessary high number of dogs affected by OA to be recruited in the RCT. It should be noted that the low placebo responder's rate (12.5% in the current study, 12.1% [81]) observed according to the objective PVF measurement again contributes to a judicious use of privately owned dogs in RCT. This is a huge advantage compared to subjective assessment completed by either veterinarians or owners, for whom the placebo responder's rate was oscillating between 25 and 44.8% [81, 82]. 

As previously demonstrated in naturally occurring OA dogs [17, 29, 31, 77] the usefulness of continuous monitoring of daily locomotor activity recording was sustained in the current study. Particularly, we denoted that continuous activity recording showed strong similarities with PVF measurement, being sensitive to functional improvement. This tool is therefore highly recommended to be used as a complement to punctual PVF measurement in a way to improve the detection of therapeutic benefits in OA dogs. In addition, the present results support the relevance of naturally occurring OA dogs for their potential to respond similarly to the human OA condition. This was illustrated in recent studies in which an anti-inflammatory drug, licofelone, was tested positively both in the dog model of experimental OA [28] naturally occurring OA [30] as well as in a clinical Phase III study in patients with knee OA [51]. Similar concordance in efficacy was observed with doxycycline [59, 60, 71]. 

Hence, dogs with the most severe limb impairment were those with the lowest degree of daily activity. This was in line with findings in human OA reporting lower physical activity in more afflicted patients [83]. Present data also give a first impression of potential benefits of an increase by 54 minutes in daily life activity being mirrored confidently with an increase in PVF that exceeded the measurement error. This was recently supported in dogs with hip OA, showing a better condition when more than an hour of exercise was performed daily [84]. Human data are also in accordance with this finding as physical activity programs are supported to reduce pain, to improve physical performance, and to delay disability among persons with knee OA [85–87].

### 4.3. Conclusion

Biomedical research and testing often faces criticism and protestation against the use of dogs for research purposes. As for any animal experiments, the three Rs' principles must apply. In addition, findings from ideal animal models have to be rapidly translated to human characteristic with the ultimate hope to better predict outcomes for human clinical trials. With this idea in mind, we present an optimization of the outcomes gained from the dog model of OA induced by CCLT. The relationship between structural changes and functional impairment denoted strong similarities with the human OA condition. This adds to the recognized anatomical and biomechanical, genomic, molecular, histological, and macroscopical structural similarities to human OA, as well as to the access of yet validated and performing functional and imaging outcome measures, as reported in the present paper.

Regarding the dog model of naturally occurring OA, the present analysis provides compelling evidence to better interpret complementary outcome measures assessing the OA condition. The PVF measurement data particularly is robust, precise, and reliable for determining whether a change has taken place as a result of an intervention. Moreover, the data support the huge interest and applicability of monitoring the level of daily locomotor activity in clinical RCT with privately-owned OA dogs. Such natural model of OA in dog represents a spontaneous model of the disease, different and complementary to the post-traumatic OA model. At the difference of the standardized preclinical CCLT dog model, the conditions are close to those of a population pharmacological study integrating, in addition to the previously listed advantages, the genomics and environmental (such as the physical activity and the nutrition) influences of the disease.

Preclinical testing protocol combining the dog model of OA induced by CCLT and the dog model of naturally occurring OA could better predict outcomes for human clinical trials in a close future, as is supported by the high translational pharmacological responsiveness.

## Figures and Tables

**Table 1:** Correlation analyses of the change in peak vertical force measurement and magnetic resonance imaging over the different phases of functional impairment before (Baseline) and following cranial cruciate ligament transection in five dogs.

	Osteophytes	Joint effusion	Focal cartilage changes	BMLs		Meniscal tears	Cartilage volume loss
				T2w-FS	T1w-GRE		
	Phase of functional impairment nadir						
*r*_*s*_	−0.05	−0.26	−0.70	−0.70	−0.70	0.01	−0.40
*P*	NS	NS	NS	NS	NS	NS	NS

	Phase of remission						
*r*_*s*_	−0.90	−0.95	−0.97	−0.70	−0.99	0.79	0.60
*P*	0.037	0.013	0.004	NS	<0.001	*P* = 0.1	*P* = 0.1

Nonsignificant at 5% level (NS).

Spearman coefficients (*r*_*s*_).

Probability value (*P*).

Bone marrow lesions (BMLs).

T1-weighted three-dimensional fast gradient recalled echo (T1w-GRE).

T2-weighted fast spin echo sequence with fat saturation (T2w-FS).

The changes in the phase of functional impairment nadir were calculated using week four values minus Baseline. The changes in the phase of remission were calculated using week 26 values minus week four.

**Table 2:** Regression analyses between the recording of the peak vertical force and macroscopic measurement of cartilage thinning at eight weeks following cranial cruciate ligament transection in 25 dogs.

Compartments			
Lateral condyle	Lateral plateau	Medial condyle	Medial plateau
NS	NS	*P* = 0.002 *m* = 0.8 [95% CI: 0.3–1.3] *b* = 0.2 [95% CI: −25.7–26.0]	NS

Nonsignificant at 5% level (NS).

Regression slope (*m*).

Regression *y*-intercept (*b*).

95% confidence intervals (95% CI).

**Table 3:** Characteristics of peak vertical force measurement in 40 privately owned dogs affected by naturally occurring osteoarthritis.

Baseline	Week 4	ICC [95% CI]	SEM	MDC_95_
56.0% BW (7.5) [26.1–66.1]	54.5% BW (8.4) [23.6–64.9]	91 [80–95]	0.7% BW	2.0% BW

Values are presented as mean (standard deviation) (minimal value–maximal value).

Intraclass coefficient of correlation (ICC).

Standard error of measurement (SEM).

Minimal detectable change at the 95% confidence level (MDC_95_).

Percentage of body weight (% BW).
